# Nobiletin Promotes Megakaryocytic Differentiation through the MAPK/ERK-Dependent EGR1 Expression and Exerts Anti-Leukemic Effects in Human Chronic Myeloid Leukemia (CML) K562 Cells

**DOI:** 10.3390/cells9040877

**Published:** 2020-04-03

**Authors:** Jui-Hung Yen, Ching-Yen Lin, Chin-Hsien Chuang, Hsien-Kuo Chin, Ming-Jiuan Wu, Pei-Yi Chen

**Affiliations:** 1 Department of Molecular Biology and Human Genetics, Tzu Chi University, Hualien 97004, Taiwan; imyenjh@hotmail.com (J.-H.Y.); jouyuan22@gmail.com (C.-Y.L.); zing910301@gmail.com (C.-H.C.); 2 Division of Cardiovascular, Department of Surgery, Kaohsiung Armed Forces General Hospital, Kaohsiung 80284, Taiwan; cvschin@gmail.com; 3 Department of Biotechnology, Chia Nan University of Pharmacy and Science, Tainan 71710, Taiwan; mingjiuanwu@gmail.com; 4 Center of Medical Genetics, Hualien Tzu Chi Hospital, Buddhist Tzu Chi Medical Foundation, Hualien 97004, Taiwan

**Keywords:** differentiation therapy, NOB, CML, megakaryocytic differentiation, EGR1

## Abstract

Differentiation therapy is an alternative strategy used to induce the differentiation of blast cells toward mature cells and to inhibit tumor cell proliferation for cancer treatment. Nobiletin (NOB), a polymethoxyflavone phytochemical, is present abundantly in citrus peels and has been reported to possess anti-cancer activity. In this study, we investigated the anti-leukemic effects of NOB on cell differentiation and its underlying mechanisms in human chronic myeloid leukemia (CML) K562 cells. NOB (100 μM) treatment for 24 and 48 h significantly decreased viability of K562 cells to 54.4 ± 5.3% and 46.2 ± 9.9%, respectively. NOB (10–100 μM) significantly inhibited cell growth in K562 cells. Flow cytometry analysis and immunoblotting data showed that NOB (40 and 80 μM) could modulate the cell cycle regulators including p21, p27, and cyclin D2, and induce G1 phase arrest. NOB also increased the messenger RNA (mRNA) and protein expression of megakaryocytic differentiation markers, such as CD61, CD41, and CD42 as well as the formation of large cells with multi-lobulated nuclei in K562 cells. These results suggested that NOB facilitated K562 cells toward megakaryocytic differentiation. Furthermore, microarray analysis showed that expression of EGR1, a gene associated with promotion of megakaryocytic differentiation, was markedly elevated in NOB-treated K562 cells. The knockdown of EGR1 expression by small interference RNA (siRNA) could significantly attenuate NOB-mediated cell differentiation. We further elucidated that NOB induced EGR1 expression and CD61 expression through increases in MAPK/ERK phosphorylation in K562 cells. These results indicate that NOB promotes megakaryocytic differentiation through the MAPK/ERK pathway-dependent EGR1 expression in human CML cells. In addition, NOB when combined with imatinib could synergistically reduce the viability of K562 cells. Our findings suggest that NOB may serve as a beneficial anti-leukemic agent for differentiation therapy.

## 1. Introduction

Chronic myeloid leukemia (CML) is a type of myeloproliferative neoplasm characterized by generating Bcr-Abl fusion oncogene, which constitutively translates into an active tyrosine kinase and leads to a massive influx of immature myeloid cells into the circulation [1,2]. Small molecule tyrosine kinase inhibitors (TKIs), such as imatinib, nilotinib, dasatinib, and bosutinib, are used as frontline therapy [3]. However, a substantial proportion of patients generated resistance or intolerance to these drugs and led to the development of second- and third-generation TKIs [4,5]. A variety of adverse effects, such as cardiovascular and metabolic toxicities, are associated with TKIs; thus, it renders the need to pursue novel therapeutic strategies [6].

Differentiation therapy is to induce the differentiation of blast cells toward a mature type and to inhibit tumor cell proliferation. This approach may devoid general cytotoxic effects, especially for the treatment of leukemia patients who cannot tolerate intensive chemotherapy or bone marrow transplantation [7]. The best example is the induction therapy of acute promyelocytic leukemia (APL) with retinoic acid (RA) and arsenic trioxide [8].

Megakaryocytes are originally derived from hematopoietic stem cells through a well-defined stepwise differentiation [9]. The bipotent erythroid/megakaryocytic progenitors can directly give rise to erythroid or megakaryocyte progenitors, which further develop into erythrocytes and megakaryocytes, respectively [10]. Megakaryocytic differentiation is an orchestrated signaling cascade that pushes cells toward cellular enlargement and polyploidization, expression of cell type-specific markers, including CD61, CD41, CD42, and other changes [11]. However, only a few transcription factors have been reported to be involved in this process, such as EGR1, GATA-1, and RUNX1 [12,13]. Previous studies of phorbol 12-myristate 13-acetate (PMA) have led to a greater understanding of the regulatory networks that participate in megakaryocytic differentiation. The pluripotent CML K562 cell line is frequently employed as a classic model to study megakaryocytic differentiation. PMA induces megakaryocytic differentiation of K562 cells by modulating protein kinase C (PKC) and the extracellular signal-regulated kinase/mitogen-activated protein kinase (ERK/MAPK) pathway, which promotes cell growth arrest, changes in cell morphology, formation of multi-lobulated nuclei, and increased expression of various genes associated with megakaryocytic differentiation [14,15].

Nobiletin (5,6,7,8,3′,4′-hexamethoxyflavone, NOB), a polymethoxyflavone (PMF), is present abundantly in the peels of certain citrus, such as sweet and mandarin oranges [16]. NOB has been demonstrated to possess a broad spectrum of bioactivities, including anti-inflammatory, anti-atherogenic, neuroprotective, and anti-tumor properties [17,18,19,20]. Moreover, the NOB shows inhibition of cell proliferation and invasion, arrest of cell cycle progression or induction of cell apoptosis in various types of solid tumors [21,22,23]. In hematological malignancies, NOB was shown to suppress cell growth and induce caspase-dependent apoptosis in human acute myeloid leukemia (AML) HL-60 cells [24]. NOB has also been identified as a modulator of cell differentiation in HL-60 cells [25]. Our previous studies showed NOB induced cell differentiation and exerted anti-leukemic effects through downregulation of the c-KIT gene in AML THP-1, U-937 and HL-60 cells [26]; however, the anti-leukemic effects of NOB in CML cells remain unclear.

In this study, we investigated whether NOB exhibits anti-cancer activities in human CML K562 cells. We evaluated the anti-leukemic effects of NOB and how it affects cancer cell proliferation, apoptosis and differentiation. We found that NOB exerted its anti-leukemic activities by inhibiting cell growth, modulating cell cycle progression and inducing K562 cells toward megakaryocyte differentiation. Moreover, we found that MAPK/ERK signaling pathway-dependent EGR1 gene expression might play a pivotal role in NOB-mediated megakaryocyte differentiation. Finally, we demonstrated a synergistic effect of the combined treatment of NOB and imatinib to reduce the viability of K562 cells. Our study provided a potential strategy by which NOB might have a crucial impact on cancer cell differentiation as a therapeutic approach in CML.

## 2. Materials and Methods

### 2.1. Chemicals

Nobiletin (NOB) was purchased from Tokyo Chemical Industry (Tokyo, Japan). Bisindolylmaleimide I (BIM) was purchased from Cayman Chemical (Ann Arbor, MI, USA). SP600125 was purchased from Enzo Life Science (Ann Arbor, MI, USA). U0126, SB203580, LY294002, 3-(4,5-dimethylthiazol-2-yl)-2,5-diphenyl tetrazolium bromide (MTT), propidium iodide, dimethyl sulfoxide (DMSO), imatinib, trypan blue and other chemicals, unless otherwise indicated, were purchased from Sigma-Aldrich Co. (St. Louis, MO, USA).

### 2.2. Cell Culture

The K562 and HEL 92.1.7 cells were obtained from the Bioresource Collection and Research Center (Hsinchu, Taiwan) and cultured in RPMI-1640 medium supplemented with 10% fetal bovine serum (FBS) (Thermo Fisher Scientific, Inc., Rockford, IL, USA), and 1% nonessential amino acids (NEAA) in a 5% CO_2_ incubator at 37 °C.

### 2.3. Measurement of Cell Viability and Cell Growth

Cells were treated with vehicle (0.1% DMSO) or NOB (10–100 μM) for 24 h or 48 h followed by incubation with MTT reagent (1 mg/mL) for 3 h at 37 °C. The cell pellets were collected and dissolved in DMSO. Cell viability was measured as the absorbance at 550 nm and according to the reduction of MTT to purple formazan crystals by mitochondria. For measurement of cell growth, cells were stained by trypan blue dye and the cell numbers were analyzed by counting of viable cells under microscopy.

### 2.4. Flow Cytometric Analysis

Cell cycle distribution was determined by flow cytometry analysis as previously described [27]. Cells were seeded on a 6-well plate and treated with vehicle or NOB for 48 h. Cells were collected and washed twice in ice-cold phosphate-buffered saline (PBS), fixed in 70% ethanol in PBS and stored at −20 °C for more than 24 h. Cells were washed in ice-cold PBS and incubated with propidium iodide (PI) staining buffer (20 μg/mL PI, 200 μg/mL RNaseA and 0.1% Triton X-100 in PBS) at room temperature for 30 min. Cell cycle distribution was measured using the FACSCalibur and analyzed by Cell Quest Pro software (BD Biosciences, San Jose, CA, USA).

The population of apoptotic cells was analyzed using an AnnexinV-FITC Apoptosis Detection Kit (Strong Biotech, Taipei, Taiwan), according to the manufacturer’s instructions. Cells were washed in ice-cold PBS, resuspended in buffer containing AnnexinV-FITC and PI, and incubated at room temperature for 15 min. The apoptotic cells were analyzed by flow cytometric analysis.

To analyze the expression of cell surface markers (CD41a, CD42a, CD42b, CD61, glycophorin A and CD71), cells were treated with vehicle or NOB (80 μM) for 48 h and incubated with a standardized panel of human specific antibody reagents to characterize populations of myeloid/megakaryocyte lineage cells as previously described [28]. Up to 50,000 cells per sample were detected by flow cytometric analysis. The levels of cell surface marker proteins were analyzed by Cell Quest Pro software and the signals from dead cells and doublets were excluded. The amounts of CD41a, CD42a, CD42b, CD61, glycophorin A, and CD71 on cell surface were expressed as the geometric mean fluorescence intensity (MFI).

### 2.5. Western Blot Analysis

Cells were treated with vehicle or NOB for the indicated periods. Total protein lysates were extracted using RIPA buffer (Thermo Fisher Scientific), and the protein concentration was measured by staining with Bradford dye reagent (Bio-Rad). Equal amounts of protein were separated by 10% SDS-PAGE, followed by transfer to polyvinylidene difluoride (PVDF) membranes. Membranes were incubated with the following antibodies: integrin subunit beta 3 (CD61), integrin subunit alpha 2b (CD41), p21 Waf1/Cip1 (p21), cyclin D2, cyclin E1, cyclin-dependent kinase 6 (CDK6), phospho-p44/42 MAP Kinase, p44/42 MAP Kinase, EGR1 (Cell Signaling Technology, Danvers, MA, USA), p27 Kip1 (p27) (ABclonal, Woburn, MA, USA), and actin (Thermo Fisher Scientific). The blots were then incubated with the appropriate horse-radish peroxidase (HRP)-conjugated secondary antibodies (Santa Cruz Biotechnology, Santa Cruz, CA, USA). Proteins were detected using Amersham ECL Prime Western Blotting Detection Reagent, and the signal was visualized on Amersham Hyperfilm ECL (GE Healthcare).

### 2.6. Quantitative Reverse Transcription-PCR (Q-RT-PCR)

Cellular RNA was prepared using an RNA Mini Kit (Geneaid, New Taipei City, Taiwan). Reverse transcription of total cellular RNA was then performed using a High-Capacity complementary DNA (cDNA) Reverse Transcription Kit (Thermo Fisher Scientific). Quantitative real-time PCR was performed in a reaction mixture containing cDNA, specific primers (Table 1) and Maxima^TM^ SYBR Green/ROX qPCR Master Mix (Thermo Fisher Scientific). Amplification was performed in a Roche LightCyclerH-480 Real-Time PCR System (Roche Diagnostics, Rotkreuz, Switzerland). PCR conditions were as follows: 50 °C for 2 min, 94 °C for 4 min, 40 cycles at 94 °C for 1 min, 58 °C for 1 min, and 72 °C for 1 min. The ∆∆C_t_ method was used for data analysis, and gene expression was estimated in triplicate samples and was normalized to GAPDH expression levels.

### 2.7. Analysis of Cell Morphology

Cells were incubated with vehicle or NOB (80 μM) for 2, 4, 6, and 8 days. Cytospins (25,000 cells) were obtained by centrifugation at 200 r.p.m. using Cytospin^TM^ 4 Cytocentrifuge (Thermo Fisher Scientific) and stained with Wright–Giemsa dye (Sigma). The cell morphology and lobulation of the nucleus were analyzed under microscopy. At least 200 cells in randomly separated fields were counted and the cells with multi-lobulated nuclei were scored and expressed as a percentage of the total cell number in these fields.

### 2.8. Microarray Analysis

Cells were treated with vehicle or NOB (80 μM) for 48 h. Total RNA was prepared using TRIzol reagent (Thermo Fisher Scientific), according to the manufacturer’s instructions. Microarray analysis was performed as previously described [26]. Briefly, Cy5-labeled aRNA was prepared for microarray hybridization to the Human One Array Plus Version 7.1 (HOA 7.1, Phalanx Biotech Group, Hsinchu, Taiwan). The fluorescence intensities of each spot were analyzed using GenePix 4.1 software (Molecular Devices, Sunnyvale, CA, USA). The normalized spot intensities were transformed to the log_2_(ratio) of gene expression, and the differentially expressed genes were established at a log_2_(ratio) ≥1.0 or ≤−1.0 and p value < 0.05 for further analysis.

### 2.9. Transfection of Small Interference RNA (siRNA) for Knockdown of EGR1 Expression

K562 cells were transfected with the siRNA negative control (si-NC) or human-specific EGR1 siRNA (Egr-1 siRNA(h):sc-29303) (Santa Cruz Biotechnology) at a final concentration of 250 pmol/mL using Lipofectamine^TM^ 2000 transfection reagent (Thermo Fisher Scientific) according to the manufacturer’s instructions. After 24 h of transfection, the cells were treated with vehicle or NOB (80 μM) for 48 h, after which cells were harvested for further analysis.

### 2.10. Analysis of Protein Kinase C (PKC) Activity

Cells were treated with NOB (80 μM) for the indicated periods. The cellular proteins were lysed in RIPA buffer, and protein kinase C (PKC) activity was determined using nonradioactive PKC activity assay kits (Enzo Life Sciences, Ann Arbor, MI, USA), according to the manufacturer’s instructions and as previously described [29]. The kinase activity was measured as the absorbance at 450 nm in a microplate reader.

### 2.11. Combination Analysis

To evaluate the pharmacological interactions of imatinib and NOB, the combination index (CI) was calculated as previously described [30]. Briefly, the CI was calculated using CI = (C_imatinib_)/(C_x_)_imatinib_+ (C_NOB_)/(C_x_)_NOB_, where (C_x_) _imatinib_ and (C_x_) _NOB_ are the doses of imatinib and NOB alone inhibiting x%; (C_imatinib_) and (C_NOB_) are the doses of imatinib and NOB in combination, which gives the experimentally observed x% inhibition. For the quantitative determination of different drug interactions, CI <1, = 1, and >1 indicate synergism, additive effect, and antagonism, respectively.

### 2.12. Statistical Analysis

All experiments were repeated at least three times, and the values were expressed as the mean ± SD. Student’s t-tests were used to assess significant differences in comparison of data with two groups. Comparison of two groups with two independent variables was performed using two-way ANOVA. Comparison of multiple groups was performed using one-way ANOVA with Dunnett’s post hoc test, and a *p* value < 0.05 was considered statistically significant.

## 3. Results

### 3.1. Effects of NOB on Cell Growth of K562 Cells

**Figure 1:**
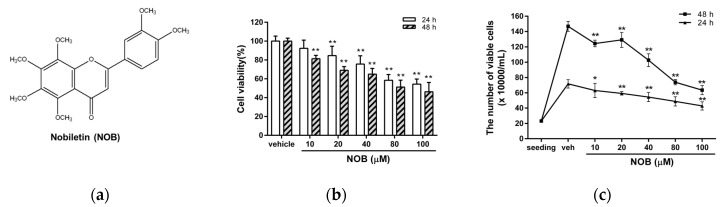
Effects of nobiletin (NOB) on the cell growth of K562 cells. (**a**) The chemical structure of nobiletin (5,6,7,8,3′,4′-hexamethoxyflavone, NOB). (**b**) K562 cells were treated with vehicle (0.1% DMSO) or NOB (10-100 μM) for 24 h or 48 h. Cell viability was measured using an MTT assay. (**c**) Cells at an initial seeding concentration of 2 × 10^5^/mL were incubated with NOB (10-100 μM) for 24 h or 48 h. The cell numbers were measured by counting of viable cells using trypan blue staining. The experiments were repeated three times. These data represent the mean ± SD of three independent experiments. *p < 0.05 and **p < 0.01 represent significant differences compared with the vehicle-treated cells (veh).

The K562 cell line is a well-known model of human chronic myeloid leukemia (CML). First, we examined the effects of nobiletin (NOB) (Figure 1a) on the viability of K562 cells. Cells were treated with vehicle or NOB for 24 h or 48 h after which cell viability was determined by MTT assay. As shown in Figure 1b, NOB significantly reduced the viability of K562 cells in a time- and dose-dependent manner. The viability of cells treated with NOB (10, 20, 40, 80, and 100 μM) for 24 h decreased to 92.4 ± 8.6%, 84.6 ± 9.9%, 75.6 ± 8.8%, 58.4 ± 6.1%, and 54.4 ± 5.3% of the control, respectively, while the viability of cells treated for 48 h decreased to 81.4 ± 3.5%, 69.1 ± 3.9%, 65.0 ± 6.1%, 51.3 ± 7.2%, and 46.2 ± 9.9% of the control, respectively (*p* < 0.01). The concentration to inhibit 50% of cell viability after 48 h treatment (IC_50_ value) was 82.49 μM. Besides, trypan blue assay was further used to reconfirm the inhibitory effect of NOB on K562 proliferation. As shown in Figure 1c, the number of viable cells was reduced and the cell proliferation was retarded in NOB-treated cells as compared to vehicle-treated group. These results suggested that NOB exhibited inhibitory effects on the cell growth of K562 cells.

### 3.2. Effects of NOB on Cell Cycle Distribution and Apoptosis of K562 Cells

**Figure 2:**
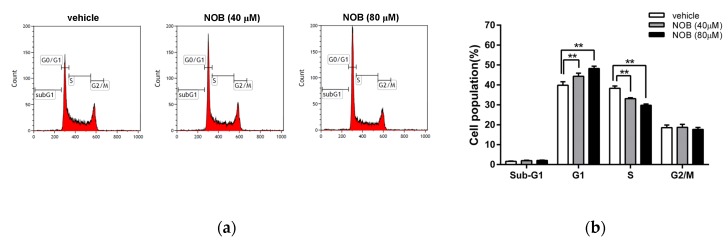

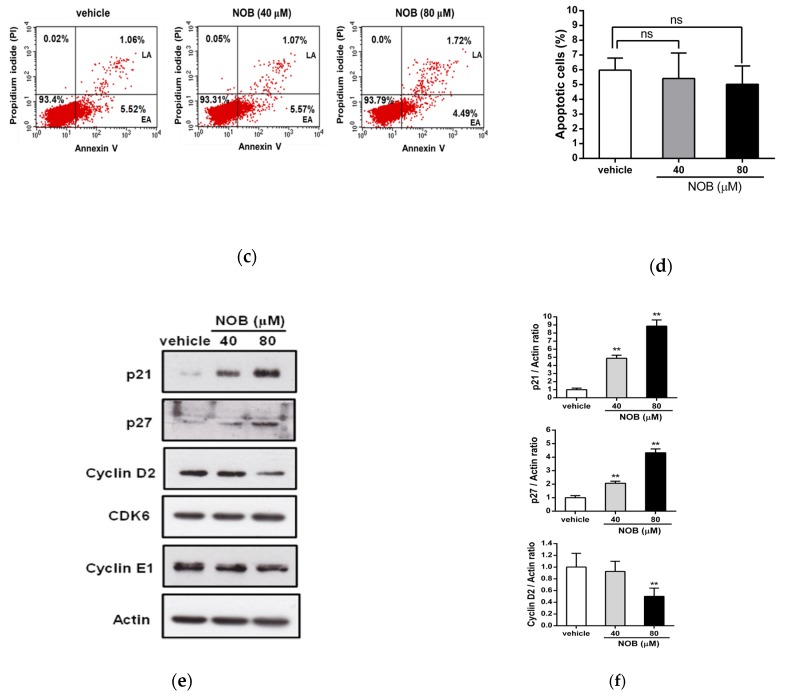
Effects of NOB on the cell cycle distribution and apoptosis of K562 cells. K562 cells were treated with vehicle or NOB (40 and 80 μM) for 24 h, and the cell distribution of cell cycle and cell apoptosis were measured by flow cytometric analysis. (**a**) A representative histogram of cell cycle distribution is shown. (**b**) The cell populations in sub-G1, G1, S, and G2/M phases were quantified. The experiments were replicated three times. These data represent the mean ± SD of independent experiments. **p < 0.01 represents significant differences compared to the vehicle-treated cells. (**c**) The population of early apoptotic (EA) and late apoptotic (LA) cells was detected using an AnnexinV-FITC and propidium iodide (PI) staining kit. A representative histogram is shown. (**d**) The percentage of apoptotic cells were measured and quantified. The experiments were replicated three times and data represent the mean ± SD of independent experiments. “ns” represents no significant differences compared to the vehicle-treated cells. (**e**) The protein expression of p21, p27, cyclin D2, CDK6, cyclin E1, and actin was measured by Western blot analysis. The immunoblots were performed at three independent experiments. A represent blot is shown. (**f**) Normalized intensities of p21, p27, and cyclin D2 protein versus actin represent the mean ± SD of three independent experiments. **p < 0.01 represents significant differences compared to the vehicle-treated cells.

We next examined whether NOB could modulate the cell cycle progression. As shown in Figure 2a,b, NOB induced a significant increase in the G1 phase cell population, which was accompanied by a decrease in cells distributed in S phases. NOB did not cause a significant change in the sub-G1 phase (population of cell death). We further examined the effect of NOB on cell apoptosis in K562 cells. As shown in Figure 2c,d, the percentage of early or late apoptotic cells was not significantly altered in NOB-treated cells as compared to the vehicle-treated group. To investigate whether NOB could modulate the cell cycle regulators for controlling the G1/S phase transition, the protein levels of p21, p27, cyclin D2, cyclin E1, and CDK6 were analyzed. As shown in Figure 2e,f, the protein levels of p21 and p27 were markedly increased, while cyclin D2 was decreased in NOB-treated K562 cells. The levels of CDK6 and cyclin E1 were not significantly changed by NOB. These results suggested that NOB suppressed cell growth through cell cycle arrest in G1 phase but did not cause significant cell death in K562 cells.

### 3.3. NOB Promoted Megakaryocytic Differentiation of K562 Cells

**Figure 3:**
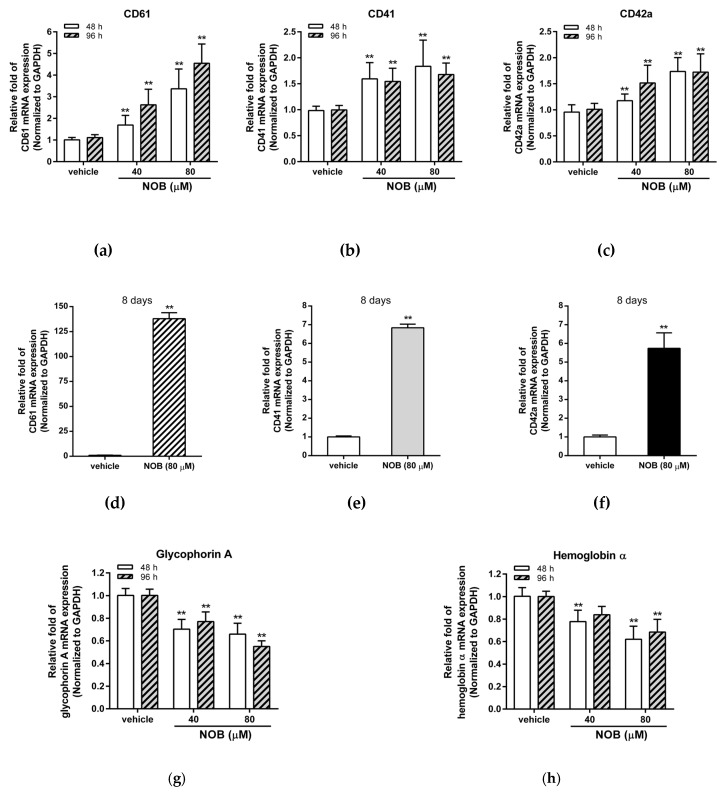
Effects of NOB on messenger RNA (mRNA) expression of cell differentiation markers in K562 cells. K562 cells were treated with vehicle or NOB (40 or 80 μM) for 48 h, 96 h, or eight days. The mRNA expression levels of (**a**,**d**) CD61 (**b**,**e**) CD41 (**c,f**) CD42a (**g**) glycophorin A (**h**) hemoglobin α were measured by Q-RT-PCR analysis and normalized to GAPDH control expression. The data represent the mean ± SD of three independent experiments. **p < 0.01 represents significant differences compared with the vehicle-treated cells.

K562 cells have been reported to differentiate into either an erythrocyte or megakaryocyte lineage depending on the inducing agents [31]. In the previous study, we had demonstrated that NOB could promote cell differentiation in various human AML cell lines [26]. Here, we investigated whether NOB could induce cell differentiation of the K562 cells. As shown in Figure 3a–c, NOB (40 and 80 μM) significantly increased the mRNA expression of megakaryocytic differentiation markers including CD61, CD41, and CD42a after NOB treatment for 48 and 96 h in K562 cells. After treatment of NOB (80 μM) for eight days, higher levels of CD61, CD41, and CD42a mRNA continued to be expressed in K562 cells (Figure 3d–f). In contrast, NOB reduced the mRNA expression of two erythrocytic marker genes, glycophorin A and hemoglobin α (Figure 3g,h). Moreover, the megakaryocytic differentiation markers induced by NOB was also observed in HEL 92.1.7 cells, another well-established human erythroleukemia cell line with properties of megakaryocytic differentiation (Figure S1a–S1c). These results indicated that NOB could increase the mRNA expression of megakaryocytic marker genes in K562 and HEL leukemia cells.

**Figure 4:**
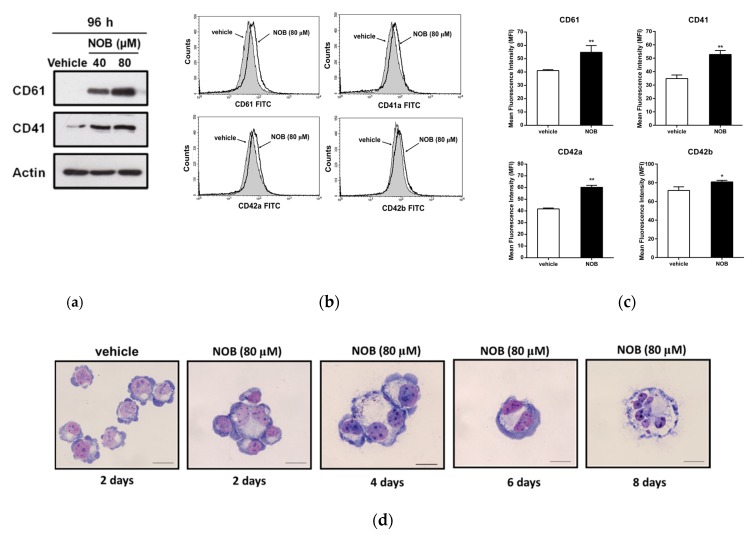
Effects of NOB on the protein expression of megakaryocytic differentiation markers in K562 cells. (**a**) Cells were treated with vehicle or NOB (40 and 80 μM) for 96 h. The protein expression of CD61, CD41 and actin was measured by Western blot analysis. The immunoblots were performed at three independent experiments. A represent blot is shown. (**b**) The amounts of cell surface protein CD61, CD41, CD42a, and CD42b were measured by flow cytometric analysis. The representative histograms are shown. (**c**) Summary of cell-surface protein levels of CD61, CD41, CD42a, and CD42b. The amounts of cell surface proteins were expressed as the geometric mean fluorescence intensity (MFI). The data represent the mean ± SD of three independent experiments. *p < 0.05 and **p < 0.01 represent significant differences compared with the vehicle-treated cells. (**d**) Cells were treated with vehicle for 2 days or NOB (80 μM) for 2, 4, 6, and 8 days and cytospins were obtained by centrifugation. The cells were stained with Wright-Giemsa dye. The cell morphological changes and multi-lobulation of the nucleus were observed under microscopy. The representative histograms are shown. Scale bar is 50μm.

We further determined the effect of NOB on the protein expression of megakaryocytic differentiation markers. As shown in Figure 4a, Western blot analysis data showed that cells treated with NOB (40 and 80 μM) for 96 h could increase the levels of CD61 and CD41 protein expression in K562 cells. Furthermore, the levels of cell-surface marker proteins in NOB-treated cells were measured by flow cytometry analysis. As shown in Figure 4b,c the protein levels of markers CD61, CD41, CD42a, and CD42b were significantly elevated by NOB (80 μM) as compared with those in the control group (*p* < 0.01). Even eight days after the NOB treatment, the CD61 or CD41 proteins were still highly expressed in K562 cells (Figure S2a–S2c). While two erythrocytic differentiation markers, glycophorin A and transferrin receptor (CD71), were reduced (Figure S3a,b). These results indicated that NOB significantly induced the expression of megakaryocytic marker proteins in K562 cells.

It is known that the megakaryocyte with multi-lobulation of the nucleus is a unique morphologic change during megakaryocytic differentiation. Thus, we examined the megakaryocytic phenotypic changes of NOB-treated K562 cells using Wright–Giemsa staining analysis. As shown in Figure 4d, NOB (80 μM) promoted the formation of large cells with multi-lobulated nuclei in K562 cells. The percentage of cells with multi-lobulated nuclei by NOB treatment for 2 to 8 days were significantly increased from 3.7% to 13.0%, compared to that of vehicle-treated group (0.9%) (Table 2). These above data revealed that NOB promoted megakaryocytic differentiation in CML cells.

### 3.4. The EGR1 Expression Induced by NOB Could Promote Megakaryocytic Differentiation of K562 Cells

**Figure 5:**
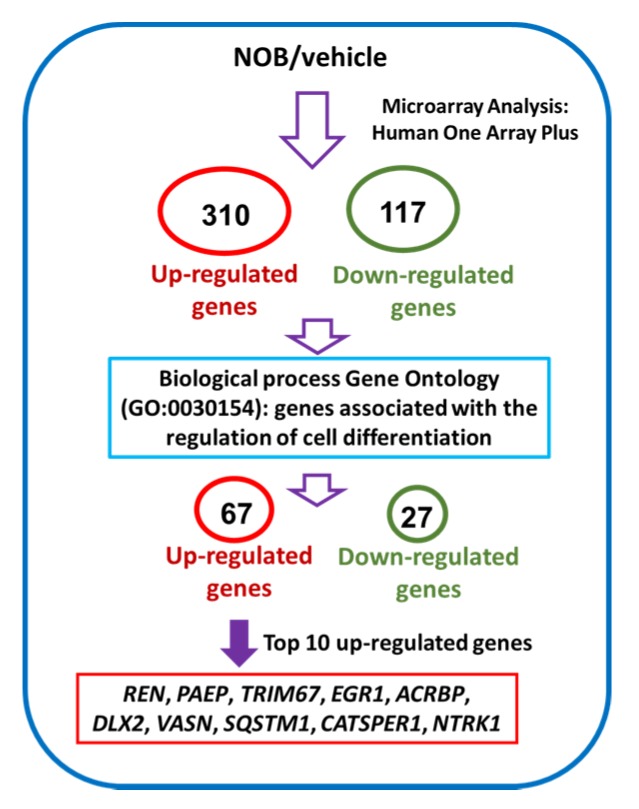
The flowchart of the microarray analysis and identification of differential gene expression in NOB-treated K562 cells.

To further explore the mechanisms how NOB mediated the megakaryocytic differentiation of K562 cells, we investigated the differential gene expression in NOB-treated cells by human genome-wide microarray analysis. K562 cells were treated with vehicle or NOB (80 μM) for 48 h, and RNA expression profiles were analyzed using the Human OneArray system. Figure 5 illustrates the flowchart for the microarray analysis to identify differentially expressed genes in NOB-treated K562 cells. A total of 310 upregulated and 117 downregulated genes were detected in cells treated with NOB compared with the vehicle-treated group. Table S1 showed that 67 upregulated and 27 downregulated genes were identified by the gene ontology enrichment analysis related to cell differentiation (GO:0030154) in NOB-treated cells.

**Figure 6:**
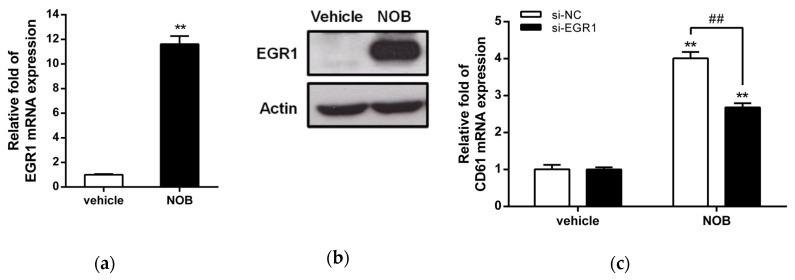
Effects of NOB on the expression of cell differentiation-related genes in K562 cells. Cells were treated with vehicle or NOB (80 μM) for 48 h. (**a**) The EGR1 mRNA levels were determined by Q-RT-PCR analysis. (**b**) The EGR1 and actin protein levels were measured by Western blot analysis. The experiments were performed at three times, and a representative blot is shown. (**c**) K562 cells were transfected with the small interference RNA (siRNA) negative control (si-NC) or si-EGR1 to knock down EGR1. The si-NC- or si-EGR1-transfected cells were treated with vehicle or NOB (80 μM) for 48 h. The level of CD61 mRNA was determined by Q-RT-PCR. The data represent the mean ± SD of three independent experiments. **p < 0.01 represents significant differences compared with the vehicle-treated cells. ##*p* < 0.01 represents significant differences compared with the si-NC-transfected group.

Table 3 showed the top 10 upregulated genes including *REN*, *PAEP*, *TRIM67*, *EGR1*, *ACRBP*, *DLX2*, *VASN*, *SQSTM1*, *CATSPER1*, and *NTRK1*. Among these genes, early growth response 1 (EGR1), a transcriptional regulator, has been reported to promote megakaryocytic and myeloid differentiation and to suppress leukemia tumorigenesis [32,33]. Then we asked whether the EGR1 gene is involved in NOB-induced megakaryocytic differentiation. As shown in Figure 6a, NOB (80 μM) treatment for 48 h increased EGR1 mRNA expression in K562 cells by approximately 11 folds when compared with the vehicle-treated group (*p* < 0.01). The EGR1 protein level was also increased by NOB (Figure 6b). To examine whether EGR1 plays an essential role during NOB-induced megakaryocytic differentiation, we examined the CD61 mRNA expression in NOB-treated K562 cells by specific siRNA knockdown of EGR1. As shown in Figure 6c, the level of CD61 mRNA in si-EGR1-transfected cells was significantly reduced by approximately 40% when compared with that of the negative control siRNA (si-NC)-transfected group in NOB-treated cells. These data indicated that knockdown of EGR1 expression attenuated NOB-induced megakaryocytic differentiation. These results suggested that NOB-induced EGR1 expression in K562 cells could drive the cells toward megakaryocytic differentiation.

### 3.5. NOB Enhances EGR1 Expression Through Activation of the MAPK/ERK Pathway

**Figure 7:**
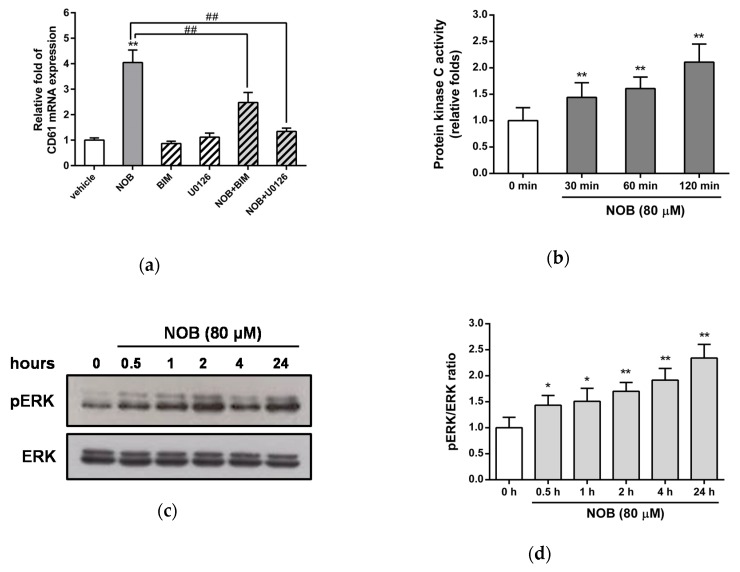

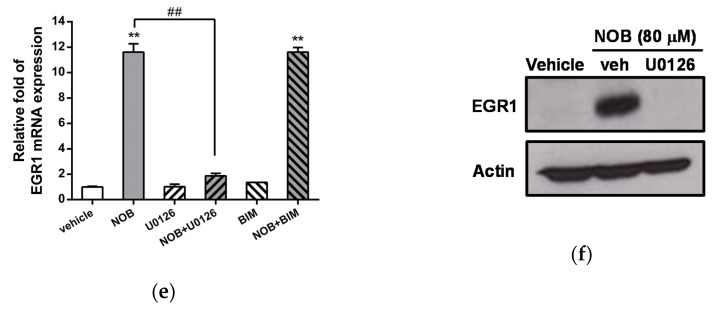
Involvement of activation of MAPK/ERK pathway dependent EGR1 expression in NOB-mediated megakaryocytic differentiation of K562 cells. K562 cells were pretreated with the protein kinase C (PKC) inhibitor Bisindolylmaleimide I (BIM) (2.5 μM) or MEK1/2 inhibitor U0126 (10 μM) for 30 min followed by treatment with NOB (80 μM) for 48 h. (**a**) The mRNA expression of CD61 was determined by Q-RT-PCR. **p < 0.01 represents significant differences compared with the vehicle-treated cells. ##*p* < 0.01 represents significant differences compared with the NOB-treated group. (**b**) K562 cells were treated with NOB (80 μM) for 30, 60 or 120 min, and PKC activity was measured by nonradioactive PKC activity assay kits. **p < 0.01 represents significant differences compared with the 0 min group. (**c**) K562 cells were treated with NOB (80 μM) for 0.5, 1, 2, 4 or 24 h, and the phosphor-ERK (pERK) and ERK protein levels were measured by Western blot analysis. A represent blot is shown. (**d**) The normalized intensity of pERK versus ERK represents the mean ± SD of three independent experiments. *p < 0.05 and **p < 0.01 represent significant differences compared with the 0 h group. (**e**) The mRNA expression of EGR1 was determined by Q-RT-PCR. **p < 0.01 represents significant differences compared with the vehicle-treated cells. ##*p* < 0.01 represents significant differences compared with the NOB alone-treated group. (**f**) The EGR1 and actin protein levels were measured by Western blot analysis. The experiments were performed in triplicate, and a representative blot is shown.

The MAPK/ERK and protein kinase C (PKC) pathways were reported to be involved in the differentiation of leukemia cells [34,35]. Thus, we further investigated whether NOB-induced megakaryocytic differentiation was dependent on activation of the PKC and MAPK/ERK pathways in K562 cells. Cells were pretreated with the PKC inhibitor bisindolylmaleimide I (BIM) or the MAPK/ERK kinase 1/2 (MEK1/2) inhibitor U0126 for 30 min followed by treatment with NOB (80 μM) for 48 h. As shown in Figure 7a, treatment of cells with BIM or U0126 alone did not significantly affect the NOB-induced CD61 mRNA expression. When K562 cells were cotreated with NOB and one inhibitor, the NOB-induced CD61 expression was significantly attenuated by BIM or U0126. The treatment of cells with the inhibitors SB203580 (p38 inhibitor), SP600125 (c-jun N-terminal kinase (JNK) inhibitor), or LY294002 (PI3K/Akt inhibitor) did not affect the NOB-induced upregulation of CD61 mRNA (Figure S3). Furthermore, we investigated the effects of NOB on the activation of PKC and the phosphorylation of ERK in K562 cells. As shown in Figure 7b, PKC activity was increased 2-fold after NOB (80 μM) treatment for 120 min compared with untreated control group. NOB treatment increased the phosphorylation of ERK1/2 protein from 0.5 h after treatment and lasted up to 24 h in K562 cells (Figure 7c,d). These results suggested that the PKC and MAPK/ERK pathways were activated in the NOB-mediated megakaryocytic differentiation of K562 cells.

We further investigated whether EGR1 expression induced by NOB was dependent on the PKC or MAPK/ERK pathways. As shown in Figure 7e, treatment of cells with BIM or U0126 alone did not affect the mRNA expression of EGR1. When K562 cells were treated with NOB in combination with U0126, but not with BIM, the NOB-induced EGR1 mRNA expression was markedly inhibited. Similarly, the EGR1 protein levels were reduced in cells that were treated with both NOB and U0126 compared with the levels in cells treated with NOB alone (Figure 7f). These data indicated that NOB promoted EGR1 gene expression through a MAPK/ERK-dependent pathway. These above results reveal that activation of the ERK-dependent EGR1 expression plays an essential role in the response to NOB-mediated CD61 marker protein expression of megakaryocyte.

### 3.6. Effects of Combined Treatment of Imatinib and NOB in K562 Cells

**Figure 8:**
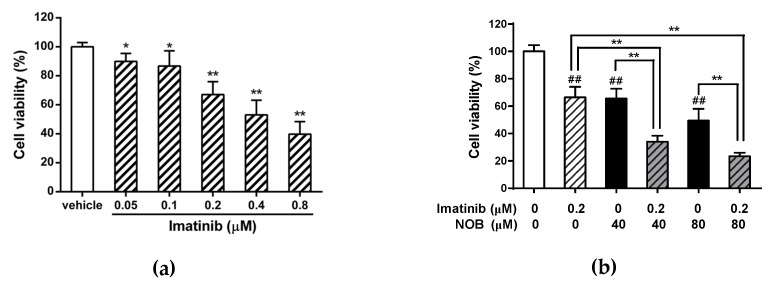
Effects of combination treatment with imatinib and NOB on cell viability of K562 cells. (**a**) K562 cells were treated with vehicle or imatinib (0.05, 0.1, 0.2, 0.4, and 0.8 μM) for 48 h. *p < 0.05 and **p < 0.01 represent significant differences compared with the vehicle-treated group. (**b**) K562 cells were treated with imatinib or NOB alone or co-treated with imatinib (0.2 μM) and NOB (40 or 80 μM) for 48 h. Cell viability was measured by MTT assay. The data represent the mean ± SD of three independent experiments. ##*p* < 0.01 represents significant differences compared with the imatinib and NOB-untreated group. **p < 0.01 represents significant differences compared with the imatinib or NOB alone groups.

We next examined the effects of the combined treatment of K562 cells with imatinib and NOB. When cells were treated with imatinib (0.05−0.8 μM) alone for 48 h, imatinib reduced cell viability in a dose-dependent manner (Figure 8a). Furthermore, K562 cells were treated with imatinib (0.2 μM) alone, NOB alone (40 or 80 μM) or both compounds for 48 h, the cells treated with a combination of imatinib and NOB showed an enhanced cytotoxicity and a reduction in viability compared with cells treated with imatinib or NOB alone (*p* < 0.01) (Figure 8b). The combination index (CI) values calculated for the combination of imatinib (0.2 μM) and NOB (40 and 80 μM) were 0.6 and 0.7 (CI < 1), respectively. These data revealed a synergistic effect of the pharmacological interaction of imatinib and NOB on reduction of cell viability. These results suggested the potential of NOB combined with imatinib to achieve a synergistic therapeutic effect in CML.

## 4. Discussion

**Figure 9:**
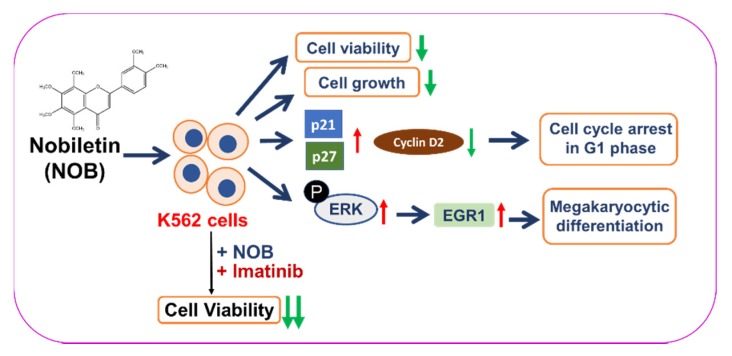
A hypothetical model for the anti-leukemic effects of NOB on human CML K562 cells. NOB reduces cell viability, inhibits cell growth, induces cell cycle arrest in G1 phase, and promotes megakaryocytic differentiation through activation of MAPK/ERK-dependent EGR1 expression in human CML K562 cells. Additionally, the combination of a conventional chemotherapeutic agent, imatinib, with NOB synergistically reduce the viability of K562 cells.

In the past four decades, several in vitro successful induction experiments have fueled the hope of developing a new approach to treat leukemia by overcoming their blocked differentiation [36,37]. In this study, we demonstrated that NOB possessed the anti-leukemic ability to reduce cell viability, inhibit cell growth, arrest cell cycle in G1 phase, and induce megakaryocyte differentiation of K562 cells. We found NOB promoted megakaryocytic differentiation through the activation of MAPK/ERK pathway-dependent EGR1 expression in K562 cells. Furthermore, we found when NOB was combined with imatinib could synergistically reduce the viability of K562 cells (Figure 9). Our findings suggest that NOB may serve as a novel therapeutic agent for patients with CML.

NOB has been reported to be nongenotoxic and to exert anti-proliferative properties in several cancer cell lines or animal cancer models [38,39,40]. Morley et al. demonstrated that NOB (60 or 100 μM) inhibited the proliferation of human breast and colon cancer cell lines by causing G1 arrest, but not cell death [41]. Ishii et al. showed that NOB strongly inhibited cell growth and influenced the cell cycle but did not efficiently induce apoptosis in human T lymphoblastoid leukemia cells (MOLT-4) [42]. In this study, NOB significantly inhibited the growth of K562 CML cells in a time- and dose-dependent manner. Our previous report showed that NOB at 100 μM had very minimal cytotoxic effects on human peripheral blood mononuclear cells (PBMCs) [26]. These studies with present results showing anti-leukemic activities are likely the most relevant biological effects of NOB. However, the viability-inhibitory ability of NOB could be very different depending on the leukemia cell type because of the higher IC_50_ value (82.49 μM) for K562 cells in the present study when compared with a lower IC_50_ value (12.6 μM) for MOLT-4 cells in another study [42].

NOB is a citrus flavonoid with multiple methoxy groups that has a poor water solubility and low oral bioavailability. This insoluble property may be relevant to low potent effects in vitro or in vivo studies. In animal studies, oral administration of NOB (50 mg/kg) in rats showed that the time to maximum concentration (t_max_) in blood was 1 h and the maximal concentrations (C_max_) of NOB in plasma was approximately 1.8 μg/mL (equivalent to 4.4 μM) [43]. After oral treatment of NOB (50 mg/kg) for 8 h in rats, NOB and its glucuronides and aglycones metabolites could be measured in plasma, the amount of NOB at this time point was approximately 2.7 μg/mL (equivalent to 6.7 μM). NOB has long half-lives, which could prolong up to 24 h in vivo [44]. In this study, we demonstrated the antileukemic effects of NOB at concentrations 10−100 μM in K562 cells, which were used in several anticancer studies in vitro, though it was higher than what can be achieved in vivo. Thus to increase the bioavailability and potent bioactivity of NOB in vivo, novel chemical modification, formulation or delivery system is needed to increase its solubility and reach the concentration we found in this study [45,46].

In this study, we found NOB could modulate cell cycle progression through arrest K562 cells in G1 phase. It is in agreement with previous findings in AML cells and in other tumor cells [26,47,48]. We also found that p21 and p27, cell cycle regulators that control G1/S transition, were significantly increased by NOB treatment; while cyclin D2 was decreased. Moreover, p21 and p27 are recognized as a cyclin-dependent kinase inhibitor and is considered one of the key regulators of cell cycle progression at G1. These findings supported that NOB has the potential to modulate the cell cycle distribution to regulate leukemia cell growth.

Megakaryocytes formation and platelets production is a complex and multi-steps processes that are characterized by expression of markers associated with megakaryocytes, alteration in cell morphology and the generation of multi-lobulated and polyploid cells through an endomitosis progression, a modified cell cycle for DNA replication without cytokinesis. Megakaryocytes then undergoes a unique maturation process to produce and release platelets [49,50]. In this study, we demonstrated that NOB significantly increased the expression of the megakaryocytic differentiation markers CD61, CD41, and CD42 in K562 cells. Moreover, NOB could increase the numbers of large cells with multi-lobulated nucleus during megakaryocytic differentiation. Even after eight days treatment of NOB, the expression of megakaryocyte markers is still highly expression; however, only about 13% of cells with multi-lobulated nucleus was detected in NOB-treated K562 cells. Through flow cytometric analysis, we found that the percentage of polyploid cells (>4N) slightly increased by NOB (but not significantly) as compared to vehicle-treated group. This data indicated that high-polyploid megakaryocytes could not appear predominately in NOB-treated cells. In bone marrow, megakaryocytes formation is regulated by cytokine signaling and several transcriptional factors for cell differentiation. Under in vitro culture condition, megakaryocytic differentiation of K562 cells could only partially mimic the physiologic progress in response to stimuli that takes place in bone marrow [51]. In this study, our in vitro finding indicated that NOB seemed to differentiate a subset of K562 into mature megakaryocytes, however, could not trigger all cells to undergo polyploidization and terminal differentiation. Whether increases of the solubility and efficacy of NOB can improve the effects on polyploidization and differentiation, or NOB can modulate cytokines in the microenvironment of bone marrow to achieve clinically relevant megakaryocytic differentiation for therapy needs further investigation.

Using microarray analysis, we discovered that upregulation of EGR1 gene expression is involved in the NOB-mediated differentiation of K562 cells. EGR1 belongs to the EGR family of C2H2-type zinc-finger proteins. It is a nuclear protein that functions as a transcriptional regulator and is required for differentiation and mitogenesis. EGR1 has been demonstrated to be a tumor suppressor involved in hematological malignancies such as AML, CML, multiple myeloma, and B cell lymphoma [52]. Studies of K562 cells revealed that PMA treatment leads to the induction of EGR1 protein in the megakaryocytic differentiation process [33,53]. Similarly, in the present study, NOB treatment also enhanced EGR1 expression at both the mRNA and protein levels. The microarray data also showed that differential mRNA expression of several genes induced by NOB that involved in regulation of cell differentiation. The functions of these genes significantly changed by NOB found in this research remain unclear and require further investigation.

It has been reported that signaling pathways affected by NOB could be linked to MAPK/ERK activity [54,55]. NOB activated the PKC-β/ε-JNK pathway but inhibited the phosphorylation of ERK1/2 to suppress tumor invasion in PMA-treated HT-1080 cells [56]. Hsiao et al. demonstrated that NOB can suppress the constitutive activity of ERK, which leads to growth arrest and apoptosis in HL-60 cells [24]. In contrast, our results appeared to suggest that the activation of ERK phosphorylation correlates with the upregulation of CD61 and this activation plays an essential role in NOB-mediated megakaryocytic differentiation. We speculated that NOB seems to induce similar effects to those of PMA, a potent PKC activator, to trigger myeloid differentiation via ERK-dependent pathways. Matsumoto et al. showed that the MAPK/ERK pathway is required for induction of p21 in leukemia cells, including THP-1, U937, and KG-1, to induce differentiation into monocytes/macrophages [35]. In K562 cells, the MAPK/ERK pathway is essential for changes of cell cycle regulators in G1 phase during PMA-induced megakaryocytic differentiation [57]. These findings as well as our results suggest that ERK phosphorylation is important to trigger the expression of genes that promote cell differentiation of human myeloid leukemia cells.

Imatinib is known to block the autophosphorylation and downstream signaling of tyrosine kinases to inhibit cellular proliferation and to trigger cancer cell apoptosis. However, TKIs are ineffective in patients who undergo blast transformation and are unable to eradicate leukemia stem cells that drive the recurrence of CML [5,6]. In clinical treatment, the highest response rates are likely achieved when new molecularly targeted therapies are combined with chemotherapy. Our results with drug combination tests showed concentration-dependent manner for the imatinib and NOB combination. Synergistic effects were demonstrated at concentrations of NOB (40 or 80 μM) and with calculated combination index of 0.6 or 0.7 (CI < 1). NOB may serve as an agent for cell differentiation and has great potential in combination with imatinib to achieve a synergistic therapeutic effect in patients with CML. In this study, we demonstrated the antileukemic effects of NOB on CML K562 cell line through inducing these cells toward megakaryocytic differentiation. We realized our studies were found only on the in vitro effect of NOB on the differentiation of CML cell line. The anti-leukemic effects of NOB on CML in animal and human studies warrant further investigation.

## 5. Conclusions

In this study, we demonstrated that NOB exerted anti-leukemic effects and promoted megakaryocytic differentiation via activation of the MAPK/ERK signaling pathway to up-regulate EGR1 gene in K562 cells. The combined treatment of NOB and imatinib exerted a notably synergistic effect and an enhanced cytotoxicity of cancer cells. Our current findings improve understanding the molecular basis of anti-leukemic activity by NOB, and provide a valuable therapeutic strategy in CML combination therapy.

## Figures and Tables

**Table 1:** The primer pairs used in real-time PCR.

Genes	Primers
CD61	5′-TGTATGGGACTCAAGATTGGA-3′ 5′-AGGGATGGCTATTAGGTTCA-3′
CD41	5′-TGCTGCTCACCATCCTGGTC-3′ 5′-AACCCAAAGCTTGGAGGCAAC-3′
CD42a	5′-TCTGTATCAGAAGCCCTGTCTTCAC-3′ 5′-GCATCGGGAGCTTTGTCTTG-3′
Glycophorin A	5′- GACAAATGATACGCACAAACGG-3′ 5′- TCCAATAACACCAGCCATCAC-3′
Hemoglobin α	5′- CAACTTCAAGCTCCTAAGCC-3′ 5′- CTTAACGGTATTTGGAGGTCAG-3′
EGR1	5′- CCGCAGAGTCTTTTCCTGAC-3′ 5′- TGGGTTGGTCATGCTCACTA-3′
GAPDH	5′- ATGAGAAGTATGACAACAGCCT-3′ 5′- AGTCCTTCCACGATACCAAAGT-3′

**Table 2:** Measurement of the cells with multi-lobulated nuclei in NOB-treated K562 cells.

	Vehicle	2 Days	4 Days	6 Days	8 Days
Cells with multilobulated nuclei (%)	0.9%	3.7%	6.9%	9.8%	13.0%

**Table 3:** The top 10 up-regulated genes associated with the regulation of cell differentiation categories based on biological process GO:0030154 in NOB-treated cells.

Gene Symbol	Description	Log_2_ (NOB/vehicle)
*REN*	renin	4.69
*PAEP*	progestagen-associated endometrial protein (PAEP)	4.42
*TRIM67*	tripartite motif containing 67	2.48
*EGR1*	early growth response 1	2.10
*ACRBP*	acrosin binding protein	1.94
*DLX2*	distal-less homeobox 2	1.82
*VASN*	vasorin	1.82
*SQSTM1*	sequestosome 1	1.80
*CATSPER1*	cation channel, sperm associated 1	1.62
*NTRK1*	neurotrophic tyrosine kinase, receptor, type 1	1.59

## Supplementary Materials

The following are available online at https://www.mdpi.com/2073-4409/9/4/877/s1, Table S1: Genes associated with the regulation of cell differentiation categories based on biological process Gene Ontology terms (GO:0030154) in NOB-treated cells; Figure S1: Effects of NOB on the mRNA expression of megakaryocytic differentiation markers in HEL cells; Figure S2. Effects of NOB on the protein expression of CD61 and CD41 in K562 cells; Figure S3: Effects of NOB on the protein expression of erythrocytic differentiation markers in K562 cells; Figure S4: Effects of NOB on kinase signaling pathways in K562 cells.
