# Supplementary material for: Nobiletin Promotes Megakaryocytic Differentiation through the MAPK/ERK-Dependent EGR1 Expression and Exerts Anti-Leukemic Effects in Human Chronic Myeloid Leukemia (CML) K562 Cells
Source: Cells. 2020 Apr 3;9(4):877. doi: 10.3390/cells9040877 (PMC7226785; doi:10.3390/cells9040877)
Supplement: Supplementary file 1 [file cells-09-00877-s001.pdf]

Supplemental Table and Figures

Table S1. Genes associated with the regulation of cell differentiation categories based on biological process Gene Ontology terms (GO:0030154) in NOB-treated cells

> GO:0030154_cell differentiation				
Entrez_gen	Gene_symbo	Description	log2(NOB/veh)	P.Value
3728	JUP	Homo sapiens junction plakoglobin (JUP)	1.015446874	0.000560126
5972	REN	Homo sapiens renin (REN)	4.688939344	0.000972917
84519	ACRBP	Homo sapiens acrosin binding protein (ACRBP)	1.935054539	0.004706668
55558	PLXNA3	Homo sapiens plexin A3 (PLXNA3)	1.009524998	0.004690686
2353	FOS	Homo sapiens FBJ murine osteosarcoma viral oncogene homolog (FOS)	1.446624408	0.016395884
23557	SNAPIN	Homo sapiens SNAP-associated protein (SNAPIN)	1.063109542	0.002845361
4669	NAGLU	Homo sapiens N-acetylglucosaminidase, alpha (NAGLU)	1.136129709	0.020508447
2539	G6PD	Homo sapiens glucose-6-phosphate dehydrogenase (G6PD)	1.370946861	0.005279236
3630	INS	Homo sapiens insulin (INS)	1.137819011	0.019503071
155465	AGR3	Homo sapiens anterior gradient 3 (AGR3)	1.213749448	0.018661039
139818	DOCK11	Homo sapiens dedicator of cytokinesis 11 (DOCK11)	1.059155715	0.011288381
50488	MINK1	Homo sapiens misshapen-like kinase 1 (MINK1)	1.134419889	0.007038157
84541	KBTBD8	Homo sapiens kelch repeat and BTB (POZ)	1.036083464	0.009487995
604	BCL6	Homo sapiens B-cell CLL/lymphoma 6 (BCL6)	1.123988358	0.006743723
8788	DLK1	Homo sapiens delta-like 1 homolog (Drosophila)	1.042075762	0.033549338
90780	PYGO2	Homo sapiens pygopus family PHD finger 2 (PYGO2)	1.125216542	0.028596043
1808	DPYSL2	Homo sapiens dihydropyrimidinase-like 2 (DPYSL2)	1.058032264	0.01342628
10628	TXNIP	Homo sapiens thioredoxin interacting protein (TXNIP)	1.08571141	0.00742551
8878	SQSTM1	Homo sapiens sequestosome 1 (SQSTM1)	1.800805079	0.010282196
3949	LDLR	Homo sapiens low density lipoprotein receptor (LDLR)	1.158016023	0.002666508
23462	HEY1	Homo sapiens hes-related family bHLH transcription factor with YRPW motif 1 (HEY1)	1.29188211	0.018298232
8367	HIST1H4E	Homo sapiens histone cluster 1, H4e (HIST1H4E)	1.511190701	0.002121614
374	AREG	Homo sapiens amphiregulin (AREG)	1.486808556	0.002382524
29967	LRP12	Homo sapiens low density lipoprotein receptor-related protein 12 (LRP12)	1.159624494	0.007691869
1958	EGR1	Homo sapiens early growth response 1 (EGR1)	2.096691475	0.000151493
4914	NTRK1	Homo sapiens neurotrophic tyrosine kinase, receptor, type 1 (NTRK1)	1.585217562	0.007705859
1745	DLX1	Homo sapiens distal-less homeobox 1 (DLX1)	1.338549848	0.014740884
89870	TRIM15	Homo sapiens tripartite motif containing 15 (TRIM15)	1.417739845	0.040336217
84498	FAM120B	Homo sapiens family with sequence similarity 120B (FAM120B)	1.332204712	0.003469503
1026	CDKN1A	Homo sapiens cyclin-dependent kinase inhibitor 1A (p21, Cip1)	1.122675687	0.002089364
595	CCND1	Homo sapiens cyclin D1 (CCND1)	1.224818015	0.006339606
5376	PMP22	Homo sapiens peripheral myelin protein 22 (PMP22)	1.18484083	0.011663354
6709	SPTAN1	Homo sapiens spectrin, alpha, non-erythrocytic 1 (SPTAN1)	1.479887278	0.010561901
4804	NGFR	Homo sapiens nerve growth factor receptor (NGFR)	1.082282607	0.002361953
1209	CLPTM1	Homo sapiens cleft lip and palate associated transmembrane protein 1 (CLPTM1)	1.208345942	0.012477485
55846	ITFG2	Homo sapiens integrin alpha FG-GAP repeat containing 2 (ITFG2)	1.425850781	0.005214441
3859	KRT12	Homo sapiens keratin 12, type I (KRT12)	1.123472358	0.000303459
124936	CYB5D2	Homo sapiens cytochrome b5 domain containing 2 (CYB5D2)	1.142792242	0.022415991
117144	CATSPER1	Homo sapiens cation channel, sperm associated 1 (CATSPER1)	1.618677386	0.001371362
348262	FAM195B	Homo sapiens family with sequence similarity 195, member B (FAM195B)	1.164210845	0.000810045
114990	VASN	Homo sapiens vasorin (VASN)	1.819085044	7.45803E-05
440730	TRIM67	Homo sapiens tripartite motif containing 67 (TRIM67)	2.482245123	0.000429643
5971	RELB	Homo sapiens v-rel avian reticuloendotheliosis viral oncogene homolog B (RELB)	1.264028218	0.000255135
9751	SNPH	Homo sapiens syntaphilin (SNPH)	1.564350272	0.003144849
1649	DDIT3	Homo sapiens DNA-damage-inducible transcript 3 (DDIT3)	1.130333498	0.002685445
10011	SRA1	Homo sapiens steroid receptor RNA activator 1 (SRA1)	1.026300324	0.031626077
1672	DEFB1	Homo sapiens defensin, beta 1 (DEFB1)	1.386701551	0.008515238
2348	FOLR1	Homo sapiens folate receptor 1 (adult)	1.129825363	0.000473935
5047	PAEP	Homo sapiens progestagen-associated endometrial protein (PAEP)	4.42333674	0.000724436
79143	MBOAT7	Homo sapiens membrane bound O-acyltransferase domain containing 7 (MBOAT7)	1.215895961	0.045702877
104	ADARB1	Homo sapiens adenosine deaminase, RNA-specific, B1 (ADARB1)	1.217802454	0.025855128
6711	SPTBN1	Homo sapiens spectrin, beta, non-erythrocytic 1 (SPTBN1)	1.103758372	0.005970047
79583	TMEM231	Homo sapiens transmembrane protein 231 (TMEM231)	1.021957524	0.0300268
4593	MUSK	Homo sapiens muscle, skeletal, receptor tyrosine kinase (MUSK)	1.346857981	0.026796274
114112	TXNRD3	Homo sapiens thioredoxin reductase 3 (TXNRD3)	1.071068576	0.025289711
91624	NEXN	Homo sapiens nexilin (F actin binding protein)	1.246404215	0.003651497
55686	MREG	Homo sapiens melanoregulin (MREG)	1.257187494	0.026804404
4208	MEF2C	Homo sapiens myocyte enhancer factor 2C (MEF2C)	1.277901364	0.002241676
30846	EHD2	Homo sapiens EH-domain containing 2 (EHD2)	1.209048781	0.036908078
2676	GFRA3	Homo sapiens GDNF family receptor alpha 3 (GFRA3)	1.032430174	0.006188036
27089	UQCRQ	Homo sapiens ubiquinol-cytochrome c reductase, complex III subunit VII, 9.5kDa (UQCRQ)	1.219189515	0.000879907
2934	GSN	Homo sapiens gelsolin (GSN)	1.198174263	0.001490973
1746	DLX2	Homo sapiens distal-less homeobox 2 (DLX2)	1.821783245	0.000824667
373863	DND1	Homo sapiens DND microRNA-mediated repression inhibitor 1 (DND1)	1.072432141	0.017361437
3275	PRMT2	Homo sapiens protein arginine methyltransferase 2 (PRMT2)	1.160195215	0.000679929
1200	TPP1	Homo sapiens tripeptidyl peptidase I (TPP1)	1.090002863	0.008265547
841	CASP8	Homo sapiens caspase 8, apoptosis-related cysteine peptidase (CASP8)	1.037888357	0.004478083
8111	GPR68	Homo sapiens G protein-coupled receptor 68 (GPR68)	-1.062694759	0.004149002
58504	ARHGAP22	Homo sapiens Rho GTPase activating protein 22 (ARHGAP22)	-1.272570436	0.000290855
5080	PAX6	Homo sapiens paired box 6 (PAX6)	-1.243246391	0.018591152
3344	FOXN2	Homo sapiens forkhead box N2 (FOXN2)	-1.398259125	0.000156594
51083	GAL	Homo sapiens galanin/GMAP prepropeptide (GAL)	-1.495179891	0.00452711
4603	MYBL1	Homo sapiens v-myb avian myeloblastosis viral oncogene homolog-like 1 (MYBL1)	-1.26383029	0.000788154
81029	WNT5B	Homo sapiens wingless-type MMTV integration site family, member 5B (WNT5B)	-1.119367746	0.015223216
3635	INPP5D	Homo sapiens inositol polyphosphate-5-phosphatase, 145kDa (INPP5D)	-1.552457686	0.019068985
3479	IGF1	Homo sapiens insulin-like growth factor 1 (somatomedin C)	-1.098545428	0.032352419
1284	COL4A2	Homo sapiens collagen, type IV, alpha 2 (COL4A2)	-1.160089869	0.018687929
2335	FN1	Homo sapiens fibronectin 1 (FN1)	-1.113676845	0.003769139
55897	MESP1	Homo sapiens mesoderm posterior basic helix-loop-helix transcription factor 1 (MESP1)	-1.167025081	0.00813489
55363	HEMGN	Homo sapiens hemogen (HEMGN)	-1.574336873	0.001393631
5979	RET	Homo sapiens ret proto-oncogene (RET)	-1.349978774	0.023284118
5176	SERPINF1	Homo sapiens serpin peptidase inhibitor, clade F (alpha-2 antiplasmin, pigment epithelium derived factor)	-1.043250123	0.002150953
948	CD36	Homo sapiens CD36 molecule (thrombospondin receptor)	-1.027043015	0.001370957
5228	PGF	Homo sapiens placental growth factor (PGF)	-1.017137361	0.026938104
660	BMX	Homo sapiens BMX non-receptor tyrosine kinase (BMX)	-2.263989688	0.000260815
4435	CITED1	Homo sapiens Cbp/p300-interacting transactivator, with Glu/Asp-rich carboxy-terminal domain, 1 (CITED1)	-1.065146762	0.001332212
3953	LEPR	Homo sapiens leptin receptor (LEPR)	-1.034431826	0.003157256
1374	CPT1A	Homo sapiens carnitine palmitoyltransferase 1A (liver)	-1.310765248	0.000586885
983	CDK1	Homo sapiens cyclin-dependent kinase 1 (CDK1)	-1.016681189	0.011218565
1830	DSG3	Homo sapiens desmoglein 3 (DSG3)	-1.035070118	0.005375953
122953	JDP2	Homo sapiens Jun dimerization protein 2 (JDP2)	-1.330424076	0.045462813
4233	MET	Homo sapiens MET proto-oncogene, receptor tyrosine kinase (MET)	-1.135425463	0.009444073
100130733	LRRC70	Homo sapiens leucine rich repeat containing 70 (LRRC70)	-1.324251354	0.002076197
729920	ISPD	Homo sapiens isoprenoid synthase domain containing (ISPD)	-1.069930476	0.023100202

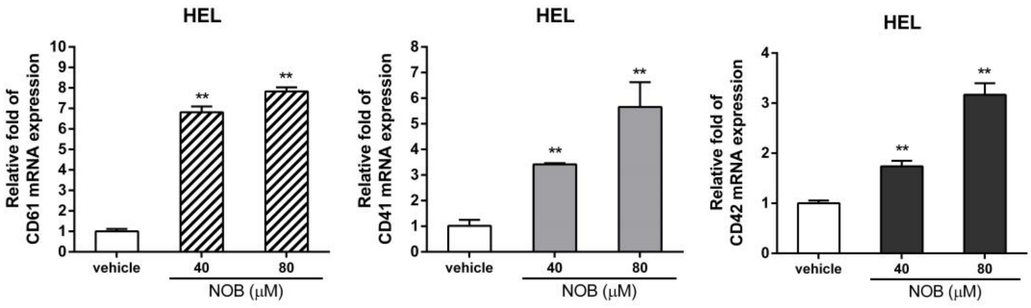

(a) **(b)** **(c)**

Figure S1. Effects of NOB on the mRNA expression of megakaryocytic differentiation markers in HEL 92.1.7 cells. The HEL cells were treated with vehicle or NOB (40 and 80 μM) for 96 h. The mRNA levels of (A) CD61 (B) CD41 (C) CD42a were measured by Q-RT-PCR analysis and normalized to GAPDH control expression.. The data represent the mean ± SD of three independent experiments. ***p*<0.01 represents significant differences compared with the vehicle-treated cells.

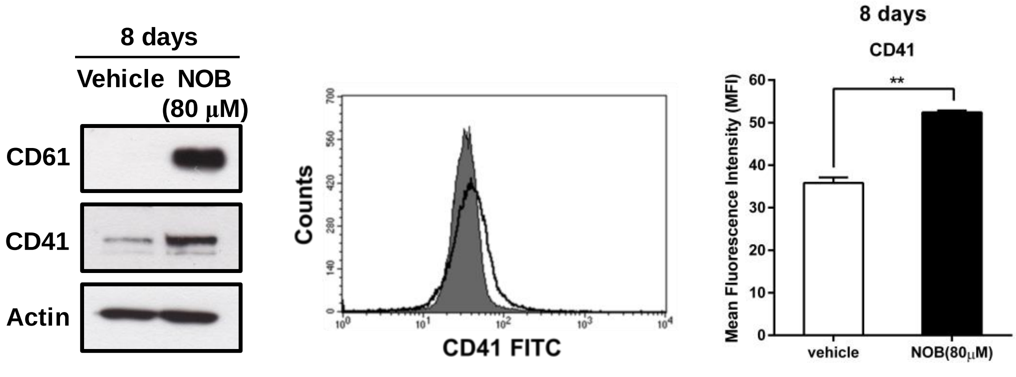

(a) (b) (c)

Figure S2. Effects of NOB treatment for 8 days on the protein expression of CD61 and CD41 in K562 cells. Cells were treated with vehicle or NOB (80 μM) for 8 days. **(a)** The protein expression of CD61, CD41 and actin was measured by Western blot analysis. The immunoblots were performed at three independent experiments. A represent blot is shown. **(b)** The level of cell-surface CD41 was measured by flow cytometric analysis. The representative histograms are shown. **(c)** The amounts of cell-surface CD41 protein was expressed as the geometric mean fluorescence intensity (MFI). The data represent the mean ± SD of two independent experiments. ** p<0.01 represent significant differences compared with the vehicle-treated cells.

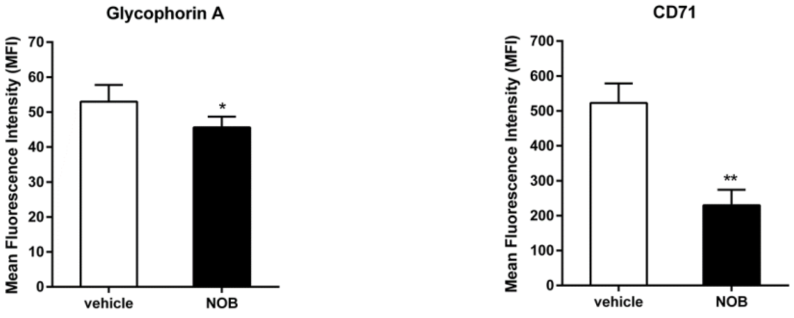

(a) **(b)**

Figure S3. Effects of NOB on the protein expression of erythrocytic differentiation markers in K562 cells. The amount of cell-surface proteins of **(a)** glycophorin A **(b)** CD71 were measured by flow cytometric analysis and the data was expressed as the geometric mean fluorescence intensity (MFI). The data represent the mean ± SD of three independent experiments. *p<0.05 and **p<0.01 represent significant differences compared to the vehicle-treated cells.

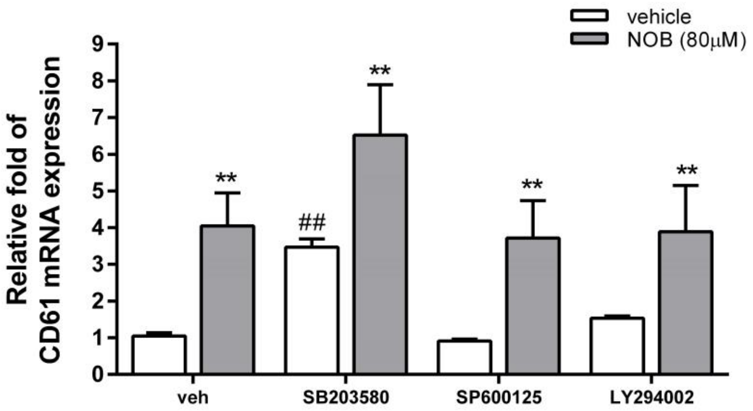

Figure S4. Effects of NOB on kinase signaling pathways in K562 cells. K562 cells were pre-treated with 0.1% DMSO (vehicle, veh) or inhibitors SB203580, SP600125 or LY294002 for 30 min followed by treatment of NOB (80 μM) for 48 h. The mRNA expression of CD 61 was determined by Q-RT-PCR. ***p*<0.01 represent significant differences compared to the inhibitor alone-treated cells. ##*p*<0.01 represents significant differences compared to vehicle alone-treated cells (veh).
